# Adhesion-clutch between DCC and netrin-1 mediates netrin-1–induced axonal haptotaxis

**DOI:** 10.3389/fnmol.2024.1307755

**Published:** 2024-02-05

**Authors:** Zhen Qiu, Takunori Minegishi, Daichi Aoki, Kouki Abe, Kentarou Baba, Naoyuki Inagaki

**Affiliations:** Laboratory of Systems Neurobiology and Medicine, Division of Biological Science, Nara Institute of Science and Technology, Nara, Japan

**Keywords:** axon guidance, haptotaxis, adhesion, clutch, netrin-1, shootin1, deleted in colorectal cancer

## Abstract

The growth cone, a motile structure located at the tip of growing axons, senses extracellular guidance cues and translates them into directional forces that drive axon outgrowth and guidance. Axon guidance directed by chemical cues on the extracellular adhesive substrate is termed haptotaxis. Recent studies reported that netrin-1 on the substrate functions as a haptotactic axon guidance cue. However, the mechanism mediating netrin-1–induced axonal haptotaxis remains unclear. Here, we demonstrate that substrate-bound netrin-1 induces axonal haptotaxis by facilitating physical interactions between the netrin-1 receptor, DCC, and the adhesive substrates. DCC serves as an adhesion receptor for netrin-1. The clutch-linker molecule shootin1a interacted with DCC, linking it to actin filament retrograde flow at the growth cone. Speckle imaging analyses showed that DCC underwent either grip (stop) or retrograde slip on the adhesive substrate. The grip state was more prevalent on netrin-1–coated substrate compared to the control substrate polylysine, thereby transmitting larger traction force on the netrin-1–coated substrate. Furthermore, disruption of the linkage between actin filament retrograde flow and DCC by shootin1 knockout impaired netrin-1–induced axonal haptotaxis. These results suggest that the directional force for netrin-1–induced haptotaxis is exerted on the substrates through the adhesion-clutch between DCC and netrin-1 which occurs asymmetrically within the growth cone.

## 1 Introduction

To establish functional neural networks, axons must navigate to appropriate synaptic partners. The growing tip of axons bears a motile structure the growth cone, which plays critical roles in axon outgrowth and guidance (Tessier-Lavigne and Goodman, 1996; Dent and Gertler, 2003; Lowery and Van Vactor, 2009; Vitriol and Zheng, 2012). Regarding the force to drive axon outgrowth and guidance, actin filaments (F-actins) polymerize at the leading edge of growth cones and disassemble proximally. This, together with the contractile activity of myosin II, drives retrograde flow of F-actin (Forscher and Smith, 1988; Katoh et al., 1999; Mallavarapu and Mitchison, 1999; Medeiros et al., 2006). Mechanical coupling between the F-actin flow and the adhesive substrate, mediated by clutch-linker molecules and cell adhesion molecules, transmits the force of F-actin flow to the substrate as traction force for growth cone migration (Mitchison and Kirschner, 1988; Suter and Forscher, 2000; Shimada et al., 2008; Giannone et al., 2009). Concurrently, the actin-adhesion coupling slows down the F-actin flow (Suter et al., 1998; Toriyama et al., 2013), allowing actin polymerization to protrude the leading-edge membrane (Mogilner and Oster, 1996).

Axon guidance is regulated by chemical cues in the extracellular environment, including soluble chemicals (termed chemotaxis) and substrate-bound chemicals (haptotaxis) (Tessier-Lavigne and Goodman, 1996; Abe et al., 2018); soluble and substrate-bound chemicals act as long-range and short-range cues, respectively (Tessier-Lavigne and Goodman, 1996). Netrin-1 is one of the best characterized axon guidance molecules (Ishii et al., 1992; Serafini et al., 1994; Lai Wing Sun et al., 2011; Boyer and Gupton, 2018); intensive research over the past decades has elucidated the molecular mechanics for netrin-1–induced axonal chemotaxis. Extracellular netrin-1 gradients, formed through diffusion, induce attraction of growth cone in vitro (Kennedy et al., 1994; Hong et al., 1999; Fothergill et al., 2014; Taylor et al., 2015; Baba et al., 2018). Stimulation of the netrin-1 receptor deleted in colorectal cancer (DCC) activates Cdc42 and Rac1 and their downstream kinase Pak1 in the growth cone (Li et al., 2002; Shekarabi and Kennedy, 2002; Shekarabi et al., 2005; Briançon-Marjollet et al., 2008; Demarco et al., 2012). Shootin1a functions as a clutch-linker molecule that links F-actin retrograde flow and adhesive substrates by interacting with the actin-binding protein cortactin and the cell adhesion molecule L1 at the growth cone (Shimada et al., 2008; Kubo et al., 2015; Baba et al., 2018). Pak1, activated through the netrin-1–induced signaling, phosphorylates shootin1a (Toriyama et al., 2013). This phosphorylation enhances its binding affinity for cortactin and L1, thereby facilitating the traction force for netrin-1–induced axon outgrowth and chemoattraction (Kubo et al., 2015; Baba et al., 2018).

Recent studies have highlighted netrin-1, present on the adhesive substrate, as a haptotactic axon guidance cue (Mai et al., 2009; Moore et al., 2009, 2012; Dominici et al., 2017; Varadarajan et al., 2017). However, the molecular mechanics of netrin-1–induced haptotaxis remains unclear (Wu et al., 2019). Here, we report that DCC and shootin1a also mediate netrin-1–induced axonal haptotaxis, through a process that does not rely on cell signaling. Shootin1a interacted with DCC, linking it to F-actin retrograde flow. Speckle imaging analyses showed that DCC underwent either grip (stop) or retrograde slip (Abe et al., 2018) on the adhesive substrate. The grip state was more prevalent on a netrin-1–coated substrate compared to the control substrate polylysine, leading to increased traction force transmission on the netrin-1–coated substrate. Furthermore, shootin1 knockout (KO) disrupted netrin-1–induced axonal haptotaxis. These findings indicate that the directional force for netrin-1–induced haptotaxis is exerted on the substrates through the asymmetric mechanical coupling between netrin-1 and DCC within the growth cone.

## 2 Materials and methods

### 2.1 Animals

All relevant aspects of the experimental procedures were approved by the Institutional Animal Care and Use Committee of Nara Institute of Science and Technology. Shootin1 KO mice were generated as described previously (Baba et al., 2018). E16.5 shootin1 KO embryos were obtained by crossing male and female shootin1 heterozygous C57BL/6 mice; the offspring genotypes were confirmed by PCR using the following primers: Genotyping F1 (5′-CAGACTGCTACCCACTACCCCCTAC-3′) and Genotyping R1 (5′-CCTAGAGCTGGACAGCGGATCTGAG-3′) for the wild-type (WT) allele. Genotyping F2 (5′-CCCAGAAAGCGAAGGAACAAAGCTG-3′), Genotyping R2 (5′-ACCTTGCTCCTTCAAGCTGGTGATG-3′) for the KO allele. Embryonic stages were calculated from noon of the vaginal plug day, defined as embryonic day 0.5 (E0.5). Chimeric mice were crossed with C57BL/6 mice for at least nine generations before analysis. All C57BL/6 mice were bred under standard conditions (12 h/12 h light/dark cycle, access to dry food and water).

### 2.2 Cell culture and transfection

Hippocampal neurons were prepared form E16.5 WT and shootin1 KO mice as described (Minegishi et al., 2021). They were cultured on glass coverslips (Matsunami) or glass bottom dishes (Matsunami), which we coated with 100 μg/mL polylysine (poly-D-lysine) (Sigma, catalog number P6407-5MG) alone or sequentially with 100 μg/mL polylysine and 400 ng/mL netrin-1 (R&D systems, catalog number 6419-N1-025/CF), and cultured in Neurobasal Medium (Thermo Fisher Scientific) containing B-27 supplement (Thermo Fisher Scientific) and 1 mM glutamine. For the immunoprecipitation analyses in Figure 3B, we used cortical neurons cultured for 2 days on polylysine-coated culture dishes (Greiner Bio-One, catalog number 664160-013) because the experiments required large numbers of neurons. Micro-scale patterns of netrin-1 on polylysine-coated coverslips were prepared as described (Abe et al., 2018), using PBS solution containing 1 M sucrose, 20 μg/ mL Texas Red-conjugated BSA (Thermo Fisher Scientific), and 400 ng/mL netrin-1 instead of 25 μg/ mL laminin. Briefly, a polydimethylsiloxane (PDMS) stamp was produced using a silicon substrate with small hexagonal patterns (Supplementary Figure 1A). PBS solution containing 1 M sucrose, 20 μg/ mL Texas Red-conjugated BSA, and 400 ng/mL netrin-1 was applied to the PDMS stamp using a micropipette to fill the indentations of microscale hexagonal lattice patterns. Excess solution on the PDMS surface was removed using a spin coater. The PDMS stamp was then attached to the polylysine-coated coverslip and incubated at 37°C overnight to coat the coverslip surface with the netrin-1 pattern. All experiments, except for force measurements, were carried out on glass surfaces. Neurons were transfected with plasmid DNA using Nucleofector (Lonza) before plating as described (Minegishi et al., 2021). HEK293T cells (ATCC, RRID:CVCL_0063) were cultured in Dulbecco’s modified Eagle’s medium (Sigma) containing 10% fetal bovine serum (FBS) (Thermo Fisher Scientific) as described previously (Baba et al., 2018), and transfected with vectors using Polyethyleneimine MAX (Polysciences) according to the manufacturer’s protocol.

### 2.3 DNA constructs

To generate pCMV-FLAG-shootin1a, the cDNA of human shootin1a was amplified by PCR with the primers Shootin1a F (5′-CCGCTCGAGATGAACAGCTCGGACGAGGAGAAG-3′) and shootin1a R (5′-CCGCTCGAGTTACTGGGAGGCCAGGATTCC CTTCAG-3′), and then subcloned into pCMV-FLAG vector (Toriyama et al., 2006). To generate pFC14K-DCC-Halotag, the cDNA of human DCC was amplified by PCR with the primers DCC F (5′-GAGGATCCATGGAGAATAGTCTTAGATG-3′), and DCC R (5′-CTGGATCCCTAAAAGGCTGAGCCTGTGATGG-3′), and then subcloned into pFC14K-Halotag CMV Flexi vector (Promega). To generate pCMV-EGFP-DCC ICD (intracellular domain), the cDNA of human DCC was amplified by PCR with the primers DCC ICD F (5′-GGTGAACTTCAAGATCCGCCACAA-3′), and DCC ICD R (5′-TGCAATAAACAAGTTAACAACAACAATTG-3′), and then subcloned into pCMV-EGFP vector (Agilent Technology). The preparation of pFN21A-HaloTag-actin has been described previously (Minegishi et al., 2018).

### 2.4 Immunocytochemistry and microscopy

Cultured neurons were fixed with 3.7% formaldehyde in Krebs buffer (118 mM NaCl, 4.7 mM KCl, 1.2 mM KH_2_PO_4_, 1.2 mM MgSO_4_, 4.2 mM NaHCO_3_, 2 mM CaCl_2_, 10 mM glucose, 400 mM sucrose, 10 mM HEPES pH 7.0) for 20 min on ice and subsequently for 10 min at room temperature, followed by treatment for 15 min with 0.05% Triton X-100 in phosphate-buffered saline (PBS) on ice and 10% FBS in PBS for 1 h at room temperature. They were then incubated with primary antibody diluted in PBS containing 10% FBS overnight at 4°C. The following primary antibodies were used: rabbit anti-shootin1a (1:2000) (Baba et al., 2018), goat anti-DCC (1:1000) (Santa Cruz Biotechnology, catalog number sc-515834), mouse anti-Tuj1 (anti-β III-Tubulin) (1:1000) (Bio Legend, catalog number 801202) and mouse anti-paxillin (1:1000) (BD Transduction Laboratories, catalog number: AB_397464) antibodies. Neurons were washed with PBS, and then incubated with secondary antibody diluted in PBS 1 h at room temperature. The following secondary antibodies were used: Alexa Fluor 488 conjugated donkey anti-goat (1:1000) (Thermo Fisher Scientific, catalog number AB_2534102), Alexa Fluor 594 conjugated donkey anti-rabbit (1:1000) (Jackson ImmunoResearch Laboratories, catalog number AB_2340621), and Alexa Fluor 488 conjugated goat anti-mouse (1:1000) (Thermo Fisher Scientific, catalog number AB_2534088) antibodies. For phalloidin staining, after washing with PBS, cells were stained with Alexa Fluor 594 conjugated phalloidin (Invitrogen, catalog number: A12381) at 1:100 dilution for 1 h at room temperature. After washing with PBS, immunostained cells were mounted with 50% (v/v) glycerol (Nacalai Tesque) in PBS. Fluorescence images were acquired using either a fluorescence microscope (BZ-X710, Keyence) equipped with 20 × 0.75 NA (Nikon) and imaging software (BZ-X viewer, Keyence) or a total internal reflection fluorescence (TIRF) microscope (IX81, Olympus) equipped with an EM-CCD camera (Ixon3, Andor), a UAPON 100 × 1.49 NA (Olympus) and imaging software (MetaMorph, Molecular Devices).

### 2.5 Immunoprecipitation and immunoblotting

For immunoprecipitation using HEK293T cells, cell lysates were prepared as described (Baba et al., 2018). The following primary antibodies were used in immunoblotting: rabbit anti-GFP (1:2000) (MBL, catalog number AB_591816) and mouse anti-FLAG (DDDDK) tag (1:4000) (MBL, catalog number AB_591224). The following secondary antibodies were used in immunoblotting: HRP-conjugated donkey anti-rabbit IgG (1:2000) (GE Healthcare, catalog number AB_772206), HRP-conjugated goat anti-mouse IgG (1:5000) (Thermo Fisher Scientific, catalog number AB_2534088). Immunoprecipitation was performed as described previously (Shimada et al., 2008; Baba et al., 2018).

For immunoprecipitation using cultured neurons, lysates of neurons were prepared as described (Baba et al., 2018), the supernatants of the lysates were incubated with rabbit anti-shootin1 antibody (Toriyama et al., 2006, 2013) or control IgG (Sigma, catalog number AB_1163661) overnight at 4°C, and immunocomplexes were then precipitated with protein G-Sepharose 4B. After washing the beads with wash buffer, immunocomplexes were analyzed by immunoblot.

The following primary antibodies were used in immunoblotting: rabbit anti-shootin1a (1:5000) (Baba et al., 2018), and mouse anti-DCC antibodies (1:4000) (Santa Cruz Biotechnology, catalog number sc-515834). The following secondary antibodies were used in immunoblotting: HRP-conjugated donkey anti-rabbit IgG (1:2000) (GE Healthcare, catalog number AB_772206), HRP-conjugated goat anti-mouse IgG (1:5000) (Thermo Fisher Scientific, catalog number AB_2534088).

### 2.6 Fluorescent speckle imaging and grip and slip analysis

Fluorescent speckle imaging and speckle tracking analysis of HaloTag-actin were performed as described in detail previously (Minegishi et al., 2021). Fluorescent speckle imaging and speckle tracking analysis of DCC-HaloTag was performed as described (Abe et al., 2018), using neurons transfected with pFC14K-DCC-Halotag. Neurons were treated with HaloTag TMR ligand (50 nM) (Promega, catalog number G8251) in culture medium and incubated for 1 h at 37°C. The ligand was then washed out with PBS, and the cells were incubated with L15 medium (Thermo Fisher Scientific) including B27 supplement (Thermo Fisher Scientific) and 1 mM glutamine for 30 min at 37°C. The fluorescent speckles of DCC-HaloTag were observed using a TIRF microscope (IX81, Olympus) equipped with an EM-CCD camera (Ixon3, Andor), a complementary metal-oxide-semiconductor (CMOS) camera (ORCA Flash4.0LT; Hamamatsu), a UAPOM 100x 1.49 NA (Olympus), and imaging software (MetaMorph, Molecular Devices). Fluorescence images were acquired every 5 s for 295 s. The flow speed of F-actin or DCC was quantified by tracing the fluorescent speckles of HaloTag-actin or HaloTag-DCC using Fiji (Open-source software package). Briefly, we selected a fluorescent speckle that flowed retrogradely for at least 20 s (4 time-frames) in a filopodium, and made a time-lapse montage including the speckle. We measured the translocation distance of the speckle; then, the flow speed during the observation was determined (Minegishi et al., 2021).

We use the terms “grip” and “slip” to describe the movement of DCC-HaloTag. “Grip vs. slip” was used by Jurado et al. (2005) to describe the degree of interaction between the cell adhesion and adhesive substrate in fish keratocytes. Subsequently, Aratyn-Schaus and Gardel (2010) used “frictional slip” to describe the experimentally observed retrograde movement of focal adhesions (including integrin) of human osteosarcoma cells on adhesive substrates. Based on their concept and terms, we previously called L1 molecules that undergo retrograde movement on adhesive substrates as in “slip” phase and those immobilized on the substrates as in “grip” phase (Abe et al., 2018, 2021); we follow this terminology here. For the grip and slip analysis, DCC puncta that were visible for at least 10 s (two intervals) were analyzed, and immobile signals were defined as DCC in grip phase while those that flowed retrogradely were defined as DCC in slip phase.

### 2.7 Traction force microscopy

Traction force microscopy was performed as described in detail previously (Minegishi et al., 2021). Neurons were transfected with pEGFP-C1 vectors and cultured on polyacrylamide (PAA) gels with embedded 200-nm fluorescent beads (Thermo Fisher Scientific) for 2 days. In this study, the PAA gel was prepared at a final concentration of 3.75% acrylamide and 0.03% bis-acrylamide. The stiffness of the PAA gel was ∼270 Pa, which is within the range of brain tissues (100-10,000 Pa) (Moore et al., 2010; Tyler, 2012; Barnes et al., 2017). Before imaging, the culture medium was replaced with L15 medium (Thermo Fisher Scientific) including B27 supplement (Thermo Fisher Scientific) and 1 mM glutamine. Time-lapse imaging of fluorescent beads and growth cone was performed at 37°C using a confocal laser microscope (LSM710, Carl Zeiss) equipped with a C-Apochromat 63x/1.2 W Corr objective, and ZEN2009 software. Images were acquired every 3 s for 147 s. After imaging, the culture dishes were treated with 1% SDS to release neurons from the PAA gel substrate, and an image of the unstrained substrate was acquired. The magnitude of the traction force under the growth cones was analyzed using MATLAB (MathWorks) and a homemade traction force analysis code (Minegishi et al., 2021).

### 2.8 Quantification and statistical analysis

Data handling and preparation of graphs was performed using Microsoft Excel 2016 (Microsoft). Statistical tests were performed using GraphPad Prism7 (GraphPad Software). For samples with more than 7 data points, the D’Agostino–Pearson normality test was used to determine whether the data followed a normal distribution. For cases in which the number of data points was between 3 and 7, the Shapiro-Wilk test was used for the normality test. We also tested the equality of variation with the F test for two independent groups that followed normal distributions. Significance tests were performed using two-tailed unpaired Student’s *t* test to compare two independent groups that followed normal distributions with equal variation. All data are shown as the mean ± SEM. Statistical significance was defined as ***p < 0.01; **p < 0.02; *p < 0.05; ns, not significant. A *P* value less than 0.05 was considered to be statistically significant. For each experiment, the corresponding statistics information and number of samples are indicated in the figure legend. All experiments were performed at least three times and reliably reproduced. Investigators were blind to the experimental groups for each analysis, except biochemical analyses. For Figures 3D, F, we measured Pearson’s correlation coefficient ’s correlation coefficient using Fiji (Open-source software package) and the JaCoP plugin (Bolte and Cordelieres, 2006) to quantify the co-localization of shootin1a with DCC and paxillin.

## 3 Results

### 3.1 Substrate-bound netrin-1 promotes traction force for axon outgrowth and haptotaxis

**FIGURE 1:**
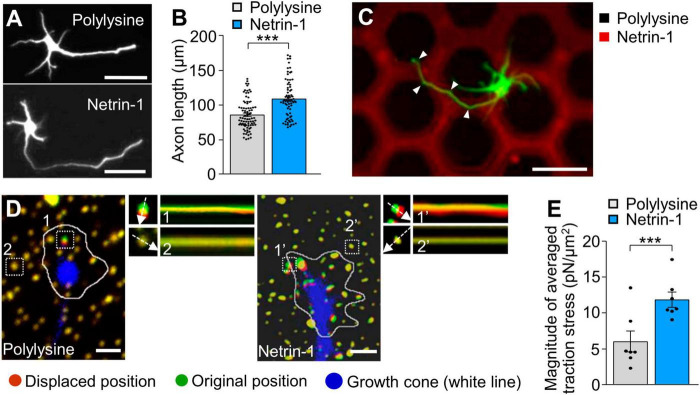
Substrate-bound netrin-1 promotes traction force for axon outgrowth and haptotaxis. **(A)** Fluorescence images of days *in vitro* (DIV) 2 hippocampal neurons cultured on glass coverslips coated with polylysine alone or coated sequentially with polylysine and netrin-1. Neurons were stained with a neuronal marker anti-Tuj1 antibody. **(B)** Quantification of axon length obtained by the analyses using neurons in panel **(A)** (polylysine, *n* = 85 cells; netrin-1, *n* = 75 cells). **(C)** A hippocampal neuron cultured on a micro-scale pattern of netrin-1 (red) and polylysine (black) for 2 days. Neurons were stained with anti-Tuj1 antibody (green). The axon turned toward netrin-1 when it reached the border between netrin-1 and polylysine (arrowheads). **(D)** Fluorescence images showing axonal growth cones of DIV 2 neurons expressing EGFP (blue) and cultured on polylysine-coated **(left)** or netrin-1–coated **(right)** polyacrylamide gel with embedded 200-nm fluorescent beads. The pictures show representative images from time-lapse series taken every 3 s for 147 s. White lines indicate the growth cone boundaries (see Supplementary Video 1). The kymographs along the axis of bead displacement (white dashed arrows) at the indicated areas show movement of beads recorded every 3 s. The beads in areas 2 and 2′ are reference beads. **(E)** Quantification of the magnitude of the traction forces under axonal growth cones on polylysine-coated or netrin-1–coated polyacrylamide gels in panel **(D)** (Polylysine = 7 growth cones; Netrin-1 = 7 growth cones). Scale bars, 25 μm for **(A)**; 50 μm for **(C)**; 2 μm for **(D)**. Data represent means ± SEM; ***p < 0.01.

To examine the effect of substrate-bound netrin-1 on axonal extension, we first cultured mouse hippocampal neurons on glass coverslips coated with either polylysine plus netrin-1 or polylysine alone as a control substrate. As reported (Dominici et al., 2017; Varadarajan et al., 2017; Wu et al., 2019), substrate-bound netrin-1 promoted axon outgrowth over the polylysine alone substrate (Figures 1A, B). In addition, neurons cultured on micro-scale patterns of netrin-1 on polylysine-coated coverslips extended axons preferentially on netrin-1 (Figure 1C), indicating that substrate-bound netrin-1 induces axon outgrowth and attraction (haptotaxis) of cultured hippocampal neurons. There was no significant difference in the sizes of axonal growth cones between on polylysine and on netrin-1 (Supplementary Figure 2). To analyze the mechanics for netrin-1–induced axon outgrowth and haptotaxis, we measured traction force produced by the axonal growth cone. Neurons were cultured on polyacrylamide gels coated with polylysine alone or polylysine plus netrin-1; 200-nm fluorescent beads were embedded in the gels. Traction force under the growth cones was analyzed by visualizing force-induced deformation of the elastic gel, which is reflected by displacement of the beads from their original positions (Minegishi et al., 2021). As reported (Chan and Odde, 2008; Toriyama et al., 2013), the beads under the growth cone moved dynamically (Figure 1D; Supplementary Video 1). The magnitude of the traction force generated on netrin-1 was 11.9 ± 1.1 pN/μm^2^ (equivalent to Pa) (*n* = 7 growth cones), which was significantly larger than on polylysine (6.1 ± 1.5 pN/μm^2^) (*n* = 7 growth cones) (Figure 1E), indicating that substrate-bound netrin-1 promotes traction force for axon outgrowth and haptotaxis.

### 3.2 Substrate-bound netrin-1 promotes F-actin-substrate coupling

**FIGURE 2:**
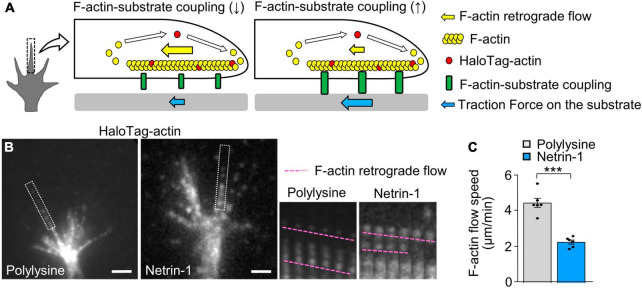
Substrate-bound netrin-1 promotes F-actin-substrate coupling. **(A)** A diagram explaining how the mechanical coupling between F-actin retrograde flow and the adhesive substrate generates traction force at the growth cone. **Right:** F-actin-substrate coupling (green) transmits the force of F-actin retrograde flow (yellow arrow) to the substrate as traction force (blue arrow) for growth cone migration. **Left:** a reduction in F-actin-substrate coupling decreases the force transmission (blue arrow), and increases the F-actin flow speed (yellow arrow) as the impediment to the flow by the coupling is diminished. **(B)** Fluorescent speckle images of HaloTag-actin in axonal growth cones cultured on polylysine **(left)** and netrin-1 **(right)**; time-lapse montages of the fluorescent speckles of HaloTag-actin in filopodia on netrin-1 and on polylysine (boxed areas) at 5-s intervals are shown to the right. F-actin flows are indicated by pink dashed lines (see Supplementary Video 2). **(C)** Quantification of F-actin flow speeds measured from the time-lapse montage analyses in panel **(B)** (polylysine, *n* = 84 signals, 6 growth cones; netrin-1, *n* = 92 signals, 6 growth cones). Scale bars, 2 μm for **(B)**. Data represent means ± SEM; ***p < 0.01.

Previous studies reported that the mechanical coupling between F-actin retrograde flow and the adhesive substrate promotes traction force (blue arrows, Figure 2A); concurrently, it reduces the F-actin flow speed (yellow arrows) (Suter and Forscher, 2000; Toriyama et al., 2013). To further examine the molecular mechanism of the netrin-1–induced force generation, we monitored F-actin retrograde flow within the axonal growth cone by fluorescent speckle imaging of HaloTag-actin (Minegishi et al., 2021). HaloTag-actin speckles moved retrogradely in growth cones (Figure 2B; Supplementary Video 2), depicting the F-actin movement (Figure 2A). The F-actin flow speed on polylysine was 4.5 ± 0.3 μm/min (mean ± SEM, *n* = 84 signals, 6 growth cones). On the other hand, netrin-l on substrates significantly reduced the flow speed (2.3 ± 0.1 μm/min, *n* = 92 signals, 6 growth cones) (Figures 2B, C; Supplementary Video 2). Together with the observed increase in traction force on netrin-1 (Figure 1E), these data indicate that netrin-1 on the adhesive substrate promotes the coupling between F-actin retrograde flow and the adhesive substrate at the growth cone.

### 3.3 DCC and shootin1a couple F-actin flow with substrate-bound netrin-1

**FIGURE 3:**
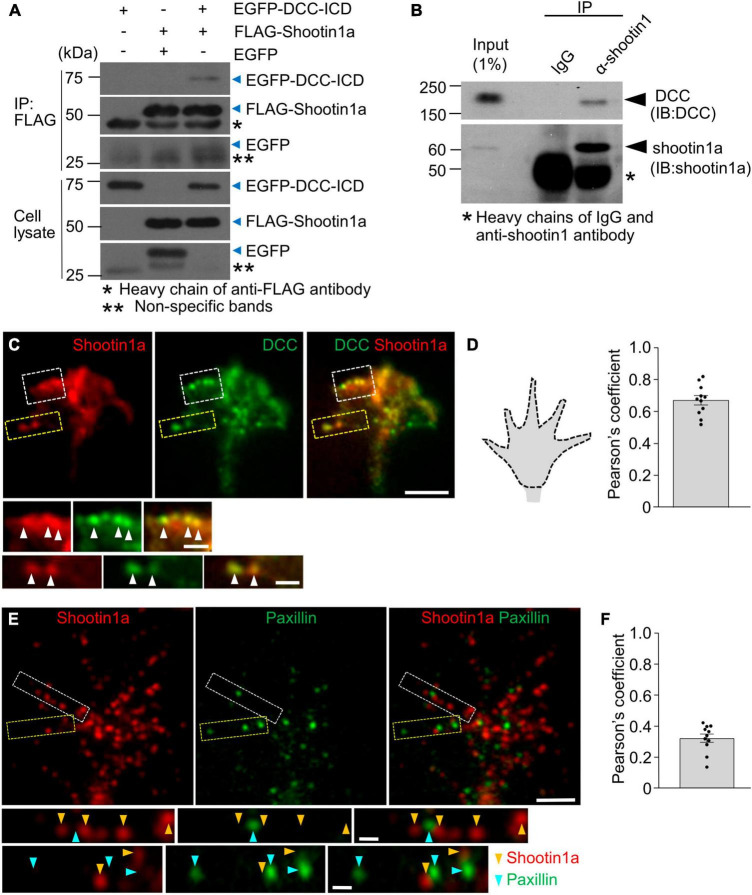
DCC interacts with shootin1a. **(A)** Co-immunoprecipitation of DCC-ICD with shootin1a in HEK 293T cells. HEK293T cells were transfected with vectors to express FLAG-shootin1a and EGFP-DCC-ICD; cells were also co-transfected with a vector to express EGFP as a negative control. Cell lysates were prepared and incubated with anti-FLAG antibody for immunoprecipitation. The immunoprecipitants were immunoblotted with anti-FLAG or anti-GFP antibody. Whole immunoblot images are presented in Supplementary Figure 3A. **(B)** Co-immunoprecipitation of endogenous shootin1a and DCC in cultured cortical neurons (DIV 2). Cell lysates were incubated with anti-shootin1 antibody or control IgG, then precipitated with protein G-Sepharose 4B. The immunoprecipitants were immunoblotted with anti-shootin1a or anti-DCC antibody. Whole immunoblot images are presented in Supplementary Figure 3B. **(C)** Fluorescence images of axonal growth cone of a DIV 2 hippocampal neuron labeled with anti-shootin1a (red) and anti-DCC (green) antibodies. Images below show enlarged views of the lamellipodium in the white square and the filopodium in the yellow square. Arrowheads indicate DCC colocalized with shootin1a. **(D)** Quantification of shootin1a-DCC colocalization using Pearson’s coefficient correlation. A typical quantified region of axonal growth cone is illustrated by the dotted line **(left)**. The Pearson’s coefficient correlations of all the growth cones (*n* = 11) exceeded 0.5 (0.68 ± 0.03), indicating colocalization of shootin1a with DCC in growth cones. **(E)** Fluorescence images of axonal growth cone of a DIV 2 hippocampal neuron labeled with anti-shootin1a (red) and anti-paxillin (green) antibodies. Images below show enlarged views of the filopodia in the squares. Yellow and cyan arrowheads indicate shootin1a and paxillin, respectively. **(F)** Quantification of shootin1a-paxillin colocalization using Pearson’s coefficient correlation. The Pearson’s coefficient correlations of all the growth cones (*n* = 11) were below 0.5 (0.33 ± 0.02), indicating weak colocalization between shootin1a and paxillin in growth cones. Scale bars: 5 μm for **(C,E)** upper figures; 1 μm for **(C,E)** enlarged figures.

Next, we examined the molecular components linking F-actin retrograde flow to the substrate-bound netrin-1. Previous studies reported that the clutch-linker molecule shootin1a interacts with the intracellular domains of the immunoglobulin superfamily adhesion molecules, L1 and N-cadherin (Baba et al., 2018; Kastian et al., 2021). This led us to hypothesize that shootin1a interacts with another member of immunoglobulin superfamily, DCC (Lai Wing Sun et al., 2011). In addition, microbead tracking analyses reported that the mechanical interaction between the growth cone and substrate-bound netrin-1 exerts forces on the substrate in a DCC dependent manner (Moore et al., 2009). To assess the involvement of DCC and shootin1a in the linkage between F-actin flow and substrate-bound netrin-1, we performed co-immunoprecipitation assay, using HEK293T cells which express shootin1a and the intracellular domain of DCC (DCC-ICD). As shown in Figure 3A, DCC-ICD was co-precipitated with shootin1a. In cell lysates prepared from cultured neurons, the full-length DCC was co-precipitated with shootin1a (Figure 3B), indicating an interaction between endogenous shootin1a and endogenous DCC in neurons. In addition, shootin1a colocalized with DCC in the axonal growth cones of hippocampal neurons (Figures 3C, D), suggesting that shootin1a interacts with DCC at growth cones. Previous studies reported integrin-dependent adhesion sites, point contacts in growth cones (Woo and Gomez, 2006). However, shootin1a did not show a marked colocalization with the point contact marker paxillin in growth cones (Figures 3E, F), suggesting that shootin1a mediates integrin-independent contacts.

**FIGURE 4:**
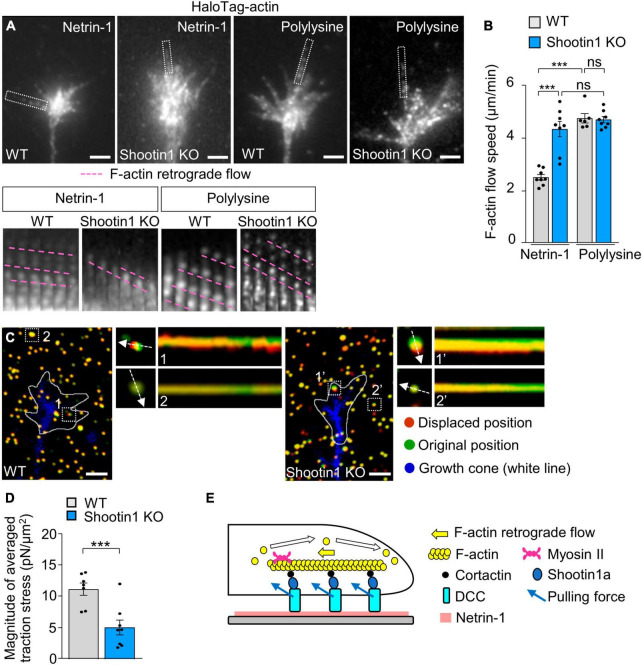
DCC and shootin1a couple F-actin flow with substrate-bound netrin-1. **(A)** Fluorescent speckle images of HaloTag-actin in axonal growth cones of WT and shootin1 KO neurons cultured on netrin-1 or polylysine. Time-lapse montages of HaloTag-actin speckles in filopodia (boxed areas) at 5-s intervals are shown below; pink dashed lines indicate the retrograde flow of speckles (see Supplementary Video 3). **(B)** Quantification of F-actin flow speeds measured from the time-lapse montage analyses in panel **(A)** (WT on netrin-1, *n* = 8 growth cones; KO on netrin-1, *n* = 8 growth cones; WT on polylysine, *n* = 6 growth cones; KO on polylysine, *n* = 8 growth cones). **(C)** Fluorescence images showing axonal growth cones expressing EGFP (blue) of DIV 2 WT **(left)** and shootin1 KO **(right)** neurons cultured on netrin-1–coated polyacrylamide gels with embedded 200-nm fluorescent beads. The pictures show representative images from time-lapse series taken every 3 s for 147 s. White lines indicate the growth cone boundaries (see Supplementary Video 4). The kymographs along the axis of bead displacement (white dashed arrows) at the indicated areas show movement of beads recorded every 3 s. The beads in areas 2 and 2′ are reference beads. **(D)** Quantification of the magnitude of the traction forces under axonal growth cones of WT and shootin1 KO neurons cultured on netrin-1–coated polyacrylamide gels in panel **(C)** (WT, *n* = 7 growth cones; shootin1 KO, *n* = 8 growth cones). **(E)** A diagram showing F-actin-substrate coupling in the axonal growth cone through cortactin, shootin1a, DCC and substrate-bound netrin-1. Shootin1a interacts with the actin binding protein cortactin (Kubo et al., 2015) and DCC (Figures 3A, B). Scale bars: 2 μm for **(A,C)**. Data represent means ± SEM; ***p < 0.01; ns, not significant.

Furthermore, disruption of shootin1a function by KO increased the F-actin flow speed in the growth cones on netrin-1–coated substrate (Figures 4A, B; Supplementary Video 3). Removal of netrin-1 from the substrate (polylysine alone) similarly increased the F-actin flow speed in WT growth cone (Figures 4A, B). In addition, shootin1a KO did not affect the F-actin flow speed in the absence of netrin-1 (polylysine alone), indicating shootin1a mediates the coupling between F-actin flow and the substrate-bound netrin-1. Concurrently, shootin1 KO reduced the magnitude of the traction force on the netrin-1–coated substrate (Figures 4C, D; Supplementary Video 4). Together, we conclude that DCC and shootin1a couple F-actin flow with substrate-bound netrin-1 (Figure 4E).

### 3.4 Netrin-1–induced axon outgrowth and haptotaxis require shootin1a-mediated actin-DCC coupling

**FIGURE 5:**
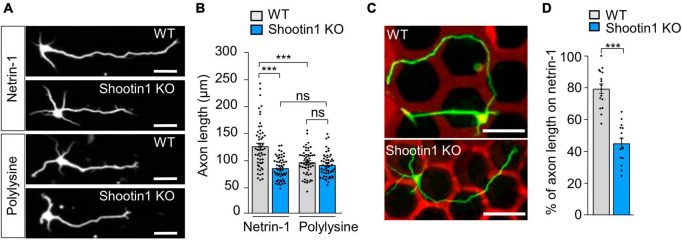
Netrin-1–induced axon outgrowth and haptotaxis require shootin1a-mediated actin-DCC coupling. **(A)** Fluorescence images of DIV 2 hippocampal neurons prepared from WT or shootin1 KO mouse and cultured on netrin-1 or polylysine. Neurons were stained with anti-Tuj1 antibody. **(B)** Quantification of axon length obtained by the analyses using neurons in panel **(A)** (WT on netrin-1, *n* = 55 growth cones; KO on netrin-1, *n* = 55 growth cones; WT on polylysine, *n* = 55 growth cones; KO on polylysine, *n* = 55 growth cones). **(C)** Hippocampal neurons cultured on micro-scale patterns of netrin-1 (red) and polylysine (black) till DIV 3. Neurons were stained with anti-Tuj1 antibody (green). **(D)** Quantification of the percentage of axon length located on netrin-1 (see Supplementary Figure 1B) (WT, *n* = 16 cells; shootin1 KO, *n* = 14 cells). Scale bars, 25 μm for **(A)**; 50 μm for **(C)**. Data represent means ± SEM; ***p < 0.01; ns, not significant.

To further examine the role of the actin-substrate linkage mediated by DCC and shootin1a, we analyzed axon outgrowth and haptotaxis. Disturbance of this linkage by shootin KO inhibited axon outgrowth on netrin-1–coated substrate (Figures 5A, B). Removal of netrin-1 from the substrate (polylysine alone) also inhibited axon outgrowth (Figures 5A, B). Consistently, shootin1a KO did not affect the axon outgrowth on the polylysine-coated substrate (Figures 5A, B). As we have seen, hippocampal neurons cultured on micro-scale patterns of netrin-1 on polylysine-coated coverslips extended axons preferentially on netrin-1 as haptotaxis (Figure 5C); 79.6% ± 3.0% (*n* = 16 neurons) of the total lengths of axons were located on netrin-1–coated substrate (Supplementary Figure 1B; Figure 5D). On the other hand, shootin1 KO neurons extended axons which frequently crossed the borders between netrin-1 and polylysine (Figure 5C); they extended significantly lower rate (45.4% ± 3.4%, *n* = 14 neurons) of axons on netrin-1–coated substrate (Figure 5D). These data indicate that netrin-1–induced axon outgrowth and haptotaxis require actin-DCC coupling mediated by shootin1a (Figure 4E).

### 3.5 Netrin-1 promotes F-actin-substrate coupling at the DCC-substrate interphase

**FIGURE 6:**
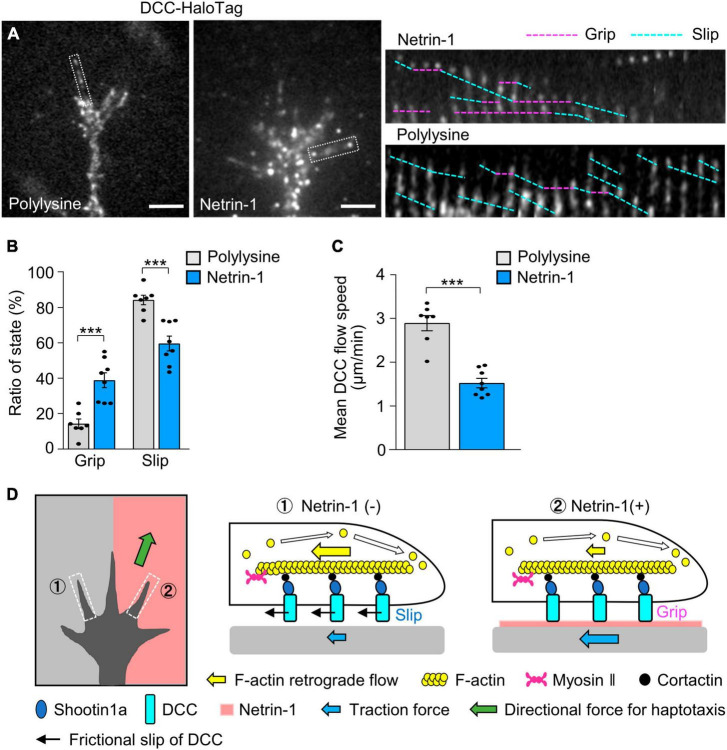
DCC in growth cones undergoes differential grip and slip on the substrates. **(A)** Fluorescent speckle images of DCC-HaloTag at the plasma membrane of axonal growth cones cultured on polylysine **(left)** and netrin-1 **(right)**; time-lapse montages of DCC-HaloTag speckles in filopodia on netrin-1 and on polylysine (boxed areas) at 5-s intervals are shown (grip and slip phases are indicated by dashed pink and blue lines, respectively) (see Supplementary Video 5). **(B,C)** Ratio of the grip and slip states **(B)** and retrograde flow speed **(C)** of DCC-HaloTag in filopodia measured from the time-lapse montage analyses in panel **(A)** (polylysine, *n* = 106 signals, 7 growth cones; netrin-1, *n* = 114 signals, 8 growth cones). **(D)** Grip and slip mechanism for netrin-1–induced axonal haptotaxis. The force of F-actin retrograde flow in the growth cone (yellow arrows) is transmitted to DCC through cortactin and shootin1a, allowing to pull the bond between DCC and the adhesive substrates. DCC molecules undergo grip or frictional slip (black arrows) on the substrates. As the substrate presenting netrin-1 has higher affinity to DCC than the control substrate, a larger number of DCC molecules undergo grip on the netrin-1-presenting substrate compared to the control substrate, leading to more efficient force transmission on the netrin-1 side (blue arrows). This asymmetric grip and slip of DCC within the growth cone generate directional force for axonal haptotaxis toward netrin-1 (green arrow). Scale bars, 2 μm for **(A)**. Data represent means ± SEM; ***p < 0.01.

To analyze the mechanism by which substrate-bound netrin-1 promotes F-actin-substrate coupling, we monitored the movement of DCC, which serves as the link between intracellular shootin1a and netrin-1 on the substrate (Figure 4E). DCC-HaloTag expressed in hippocampal neurons was labeled with TMR ligand and observed using a TIRF microscope (Figure 6A; Supplementary Video 5). Two distinct types of DCC signals were observed: retrogradely flowing (blue dashed lines) and immobile (pink dashed lines, Figure 6A) signals. We previously reported similar movements of L1 at the growth cone during laminin-induced axonal haptotaxis (Abe et al., 2018) and mechanosensing (Abe et al., 2021). At the growth cone, the force of F-actin retrograde flow is transmitted to L1 through cortactin and shootin1a, allowing to pull the bond between L1 and laminin. When the pulling force exceeds a threshold, the bond breaks and L1 flows retrogradely on the substrate (Abe et al., 2018, 2021).

We expect that similar force are transmitted to DCC, thereby pulling the bond between DCC and substrate-bound netrin-1 (blue arrows, Figure 4E). We refer to DCC that undergoes retrograde movement on the substrates as being in “slip” phase and that immobilized on the substrates as being in “grip” phase, following the terminology of L1 movements (see Materials and Methods) (Abe et al., 2018). On polylysine, 15% of DCC speckles exhibited the grip phase, while 85% displayed slip phase (*n* = 350 speckles, 7 growth cones). In contrast, 40% of the signals were in grip phase on netrin-1 (*n* = 548 speckles, 8 growth cones) (Figure 6B). The grip phase percentage was significantly larger on netrin-1 than on polylysine, indicating that DCC binds to the netrin-1–coated substrate with higher affinity. Consistently, the mean flow speed of DCC on the netrin-1–coated substrate was 1.63 ± 0.11 μm/min (*n* = 114 signals, 8 growth cones), significantly slower than that on the polylysine-coated substrate (3.09 ± 0.18 μm/min, *n* = 106 signals, 7 growth cones) (Figure 6C). Together, these data suggest that netrin-1 promotes F-actin-substrate coupling at the interphase between DCC and the substrate, thereby generating traction forces for axon outgrowth and haptotaxis (Figure 6D).

## 4 Discussion

### 4.1 Axonal haptotaxis through adhesion-clutch between DCC and netrin-1

We have shown that DCC and shootin1a mechanically couple F-actin retrograde flow in the axonal growth cone and netrin-1 on the adhesive substrate. Substrate-bound netrin-1 increased the grip phase of DCC and promoted F-actin-substrate coupling, thereby increasing traction force produced by the growth cone. Furthermore, inhibition of the mechanical coupling by shootin1a KO disrupted netrin-1–induced axon outgrowth and haptotaxis. These data suggest a mechanism by which the growth cone generates a directional force for netrin-1–induced axonal haptotaxis (Figure 6D). The force of F-actin retrograde flow in the growth cone (yellow arrows) is transmitted to DCC through cortactin and shootin1a, allowing to pull the bond between DCC and the adhesive substrates. When the pulling force exceeds a threshold, the bond breaks and DCC molecule flows retrogradely on the substrate (black arrows). As the substrate presenting netrin-1 exhibits higher affinity to DCC than the control substrate, more DCC molecules grip the netrin-1-presenting substrate than the control substrate, enabling more efficient force transmission on the netrin-1 side (blue arrows). This asymmetric adhesion-clutch between DCC and netrin-1 within the growth cone generate directional force for axonal haptotaxis toward netrin-1 (green arrow).

Our data suggest that shootin1a mediates integrin-independent contacts at the growth cone (Figures 3E, F). Speckle imaging data of DCC provide key information on the adhesion dynamics of this system. It has been reported that the frictional slip between integrins and the substrate decreases during the maturation of integrin-mediated focal adhesions (Aratyn-Schaus and Gardel, 2010). On the other hand, DCC repeats immobilized phase (grip) and frictional slip phase (slip) on the netrin-1–coated substrate (Figures 6A–C). We have also observed repeated grip and slip phases of the L1-shootin1a adhesion-clutch under various elastic conditions that correspond to the soft brain environments (Abe et al., 2021). These data suggest that the bond between DCC and the adhesive substrate breaks repeatedly, without undergoing maturation. As maturation of the adhesion machinery promotes formation of a strong adhesion that can inhibit cell migration (Palecek et al., 1997; Liu et al., 2015), we consider that the slippery adhesion-clutch between DCC and the substrate is suitable for the efficient growth cone migration.

### 4.2 Adhesion-clutch mechanism requires a specific adhesion ligand on the substrate

The present study showed that the removal of netrin-1 from the substrate (polylysine alone) resulted in an increase in the F-actin flow speed in growth cones, indicating that the netrin-1 removal disrupts the actin-adhesion coupling (Figure 4B). Consistently, shootin1a KO did not affect F-actin retrograde flow or axon outgrowth in the absence of netrin-1 on the substrate (Figures 4A, B, 5A, B). These data demonstrate that the adhesion-clutch mechanism requires a proper adhesion ligand on the substrate and emphasize the importance of conducting experiments using appropriate adhesion ligands. There is a debate regarding the contribution of the adhesion-clutch mechanism to axon growth in 3D environments. Santos et al. (2020) reported that disruption of F-actin by cytochalasin D treatment promoted the extension of the longest neurite on the polylysine-coated substrate. Together with the data that they could not detect neuronal traction force in 3D environments, Santos et al. (2020) concluded that axon outgrowth in 3D is independent of adhesions. However, the cytochalasin D treatment inhibited extension of the longest neurite on a laminin-coated substrate as described in Supplementary Figure 9 of Toriyama et al. (2010). As laminin serves as a proper substrate for the shootin1a-L1 adhesion-clutch (Abe et al., 2018), the data of Toriyama et al. (2010) indicate that F-actin is involved in axon outgrowth on the appropriate substrate.

Furthermore, in a semi-3D environment, Minegishi et al. (2018) detected traction force produced by the growth cone located at the leading process of migratory neurons. The force of about 10 pN/μm^2^ was produced by shootin1-L1 adhesion-clutch. Importantly, shootin1 KO reduced the force generation as well as neuronal migration speed in brain tissues. Furthermore, shootin1 KO reduced axonal extension in brain (Baba et al., 2018). Together, these reported data suggest that neuronal growth cones can produce pulling force to propel their migration in 3D environments. In addition to shootin1, catenins (Bard et al., 2008; Garcia et al., 2015), ezrin/radixin/moesin (ERM) proteins (Sakurai et al., 2008; Marsick et al., 2012), vinculin (Lamoureux et al., 1989; Varnum-Finney and Reichardt, 1994; Sydor et al., 1996; Turney et al., 2016; Mandal et al., 2021), talin (Lamoureux et al., 1989; Sydor et al., 1996; Wang et al., 2021), and fmn2 (Ghate et al., 2020) have been proposed as clutch-linker molecules for growth cone advance.

### 4.3 DCC-shootin1a as a dual-mode regulator of netrin-1–induced axon guidance

The present axon guidance mechanism does not rely on cell signaling but depends on differences in the physical force between DCC and the adhesive substrates, with DCC serving as an adhesion receptor for netrin-1. This mechanism is consistent with the previous report that growth cones exerted a pulling force on netrin-1 in a DCC-dependent manner (Moore et al., 2009). Additionally, we recently reported a similar mechanism for laminin-induced axonal haptotaxis, where asymmetric adhesion-clutch between the adhesion receptor L1 and the substrate mediates axon turning (Abe et al., 2018). Given its simplicity, we consider that this mechanism may contribute to various haptotactic axon guidance and cell migration. Versatility of this mechanism in haptotactic cell motility is an intriguing issue for further investigation.

DCC also serves as a cell signaling receptor for netrin-1 (Lai Wing Sun et al., 2011). Netrin-1 binding to DCC activates Cdc42, Rac1 and Pak1 (Li et al., 2002; Shekarabi and Kennedy, 2002; Shekarabi et al., 2005; Briançon-Marjollet et al., 2008; Demarco et al., 2012; Boyer and Gupton, 2018). Netrin-1–induced axonal chemoattraction is also mediated by various signaling molecules, including phosphatidylinositol-3 kinase, focal adhesion kinase, phospholipase Cγ, cAMP, Ca^2+^, ERK1/2, and Src (Song and Poo, 2001; Gomez and Zheng, 2006; Lowery and Van Vactor, 2009; Lai Wing Sun et al., 2011; Moore et al., 2012; Gomez and Letourneau, 2014; Sutherland et al., 2014). In addition, shootin1a mediates netrin-1–induced axon guidance under the DCC signaling cascade; it is phosphorylated by Pak1 upon DCC stimulation and dephosphorylated by protein phosphatase-1 (PP1) (Toriyama et al., 2013; Kastian et al., 2023). Phosphorylation of shootin1a enhances the clutch-coupling between F-actin and the adhesion receptor L1 and promotes the traction force for netrin-1–induced axon outgrowth and chemoattraction (Kubo et al., 2015; Baba et al., 2018). Netrin-1 gradients induce chemoattraction even without substrate-bound netrin-1, indicating that netrin-1 can function as a chemotactic guidance cue (Baba et al., 2018). The mechanism involving shootin1a phosphorylation is highly sensitive to extracellular gradients of netrin-1 (Baba et al., 2018), potentially involving desensitization and re-adaptation of the signaling pathway for long-distance axon guidance (Kastian et al., 2023). During navigation, growth cones may sense netrin-1 as a short-range adhesion cue or a long-range cell signaling ligand, accommodating to different environments along their routes, to travel to their destinations.

## Data availability statement

The original contributions presented in this study are included in this article/Supplementary material, further inquiries can be directed to the corresponding author.

## Ethics statement

The animal study was approved by the Institutional Animal Care and Use Committee of Nara Institute of Science and Technology. The study was conducted in accordance with the local legislation and institutional requirements.

## Author contributions

ZQ: Data curation, Formal analysis, Methodology, Investigation, Validation, Visualization, Writing – original draft, Writing – review and editing. TM: Funding acquisition, Methodology, Supervision, Validation, Writing – original draft, Writing – review and editing. DA: Data curation, Formal analysis, Investigation, Writing – review and editing. KA: Methodology, Supervision, Writing – review and editing. KB: Data curation, Investigation, Methodology, Validation, Writing – review and editing. NI: Conceptualization, Funding acquisition, Project administration, Supervision, Validation, Writing – original draft, Writing – review and editing.

## Supplementary material

Supplementary Video 1 Time-lapse movies of axonal growth cones of hippocampal neurons expressing EGFP (blue) and cultured on polylysine-coated and netrin-1–coated polyacrylamide gels with embedded 200-nm fluorescent beads (see Figure 1D). Images were acquired every 3 s for 147 s using a confocal microscope.

Supplementary Video 2 Time-lapse movies of HaloTag-actin in axonal growth cones of hippocampal neurons cultured on polylysine-coated and netrin-1–coated glass-bottom dishes (see Figure 2B). Images were acquired every 5 s for 295 s using a fluorescence microscope.

Supplementary Video 3 Time-lapse movies of HaloTag-actin in axonal growth cones of a WT hippocampal neuron and a shootin1 KO hippocampal neuron cultured on netrin-1–coated glass-bottom dishes (see Figure 4A). Images were acquired every 5 s for 295 s using a fluorescence microscope.

Supplementary Video 4 Time-lapse movies of axonal growth cones of a WT hippocampal neuron and a shootin1 KO hippocampal neuron expressing EGFP (blue) and cultured on netrin-1–coated polyacrylamide gels with embedded 200-nm fluorescent beads (see Figure 4C). Images were acquired every 3 s for 147 s using a confocal microscope.

Supplementary Video 5 Time-lapse movies of DCC-HaloTag in axonal growth cones of hippocampal neurons cultured on polylysine-coated and netrin-1–coated glass-bottom dishes (see Figure 6A). Images were acquired every 5 s for 245 s using a TIRF microscope.

Supplementary Figure 1 Analysis of netrin-1–induced axonal haptotaxis. **(A)** Preparation of micro-scale patterns of netrin-1 on polylysine-coated coverslips. For details, see Materials and Methods. **(B)** Analysis of axons located on netrin-1. To calculate % of axon length on netrin-1, the total lengths of axonal shafts located on netrin-1–coated areas (blue double arrow) were divided by the full lengths of the axonal shafts (black double arrow).

Supplementary Figure 2 Analysis of the size of axonal growth cone on netrin-1 and polylysine. **(A)** Fluorescence images of axonal growth cones of DIV2 hippocampal neurons cultured on glass coverslips coated with polylysine alone or coated with polylysine and netrin-1. Neurons were stained with Alexa Fluor 594 conjugated phalloidin for F-actin. **(B)** Quantification of growth cone size obtained by the analyses using neurons in panel **(A)** (polylysine, *n* = 40 growth cones; netrin-1, *n* = 40 growth cones). Scale bars, 5 μm. Data represent means ± SEM; ns, not significant.

Supplementary Figure 3 Whole immunoblot images in Figures 3A, B. **(A)** HEK293T cells were transfected with vectors to express FLAG-shootin1a and EGFP-DCC-ICD; cells were also co-transfected with a vector to express EGFP as a negative control. Cell lysates were prepared and incubated with anti-FLAG antibody for immunoprecipitation. The immunoprecipitants were immunoblotted with anti-FLAG or anti-GFP antibody. **(B)** Cortical neurons prepared from E16.5 WT mice were seeded on polylysine-coated dishes and cultured for 2 days. Cell lysates were prepared and incubated with anti-shootin1 antibody or control IgG, then precipitated with protein G-Sepharose 4B. The immunoprecipitants were immunoblotted with anti-shootin1a or anti-DCC antibody.
